# Urinary antibiotic activity in paediatric patients attending an outpatient department in north-western Cambodia

**DOI:** 10.1111/tmi.12398

**Published:** 2014-10-16

**Authors:** Katherine R W Emary, Michael J Carter, Sreymom Pol, Soeng Sona, Varun Kumar, Nicholas P J Day, Christopher M Parry, Catrin E Moore

**Affiliations:** 1 Mahidol-Oxford Tropical Medicine Research Unit, Faculty of Tropical Medicine, Mahidol UniversityBangkok, Thailand; 2 Centre for Clinical Vaccinology and Tropical Medicine, Nuffield Department of Clinical Medicine, University of Oxford, Churchill HospitalOxford, UK; 3 Angkor Hospital for ChildrenSiem Reap, Cambodia; 4 Institute of Child Health, University College LondonLondon, UK; 5 Clinical Sciences, Liverpool School of Tropical MedicineLiverpool, UK

**Keywords:** antibiotics, paediatric, Cambodia

## Abstract

**Objective:**

Antibiotic resistance is a prominent public and global health concern. We investigated antibiotic use in children by determining the proportion of unselected children with antibacterial activity in their urine attending a paediatric outpatient department in Siem Reap, Cambodia.

**Methods:**

Caregiver reports of medication history and presence of possible infection symptoms were collected in addition to urine samples. Urine antibiotic activity was estimated by exposing bacteria to urine specimens, including assessment against multiresistant bacteria previously isolated from patients in the hospital (a methicillin-resistant *Staphylococcus aureus* (MRSA), a multiresistant *Salmonella typhi* and an extended-spectrum *β*-lactamase (ESBL)-producing *Escherichia coli* isolate).

**Results:**

Medication information and urine were collected from 775 children. Caregivers reported medication use in 69.0% of children in the preceding 48 h. 31.7% samples showed antibacterial activity; 16.3% showed activity against a local multiresistant organism. No specimens demonstrated activity against an ESBL-producing *E. coli*.

**Conclusions:**

Antibiotics are widely used in the community setting in Cambodia. Parents are often ill-informed about drugs given to treat their children. Increasing the regulation and training of private pharmacies in Cambodia may be necessary. Regional surveillance of antibiotic use and resistance is also essential in devising preventive strategies against further development of antibiotic resistance, which would have both local and global consequences.

## Introduction

There is global concern regarding the rising level of antimicrobial resistance (WHO 2014). High levels of antimicrobial resistance have been demonstrated in South-east Asia amongst *Enterobacteriaceae* (Lu *et al*. 2012), *Streptococcus pneumoniae* (Kim *et al*. 2012), *Salmonella typhi* (Parry & Threlfall 2008) and *Staphylococcus aureus* (Song *et al*. 2011); multiple factors contribute to resistance, including poor health infrastructure and the widespread, often inappropriate, use of antibiotics in medicine and agriculture. In many developing countries such as Cambodia, the sale of antibiotics is unregulated and requires neither a medical consultation nor a prescription. It is therefore likely that antibiotics are widely used in the community, and this may contribute to the bacterial resistance found in Cambodia (Chheng *et al*. 2009, 2013; Emary *et al*. 2012) particularly if taken at subtherapeutic doses and for inadequate durations (Morgan *et al*. 2011). Moreover in a paediatric patient, the use of antibiotics is often inappropriate as many febrile illnesses are due to viruses (Hersh et al., 2011) and the prescription for an antibiotic may cause more harm than good due to short term side effects and long term consequences such as the development of resistance (Costelloe *et al*. 2010). There are guidelines to aid clinicians in the use of antibiotics in resource-limited settings. The Integrated Management of Childhood Illness Handbook (WHO 2005) is designed for use in the absence of many diagnostic facilities and describes clinical indications where antibiotic use is appropriate, such as pneumonia, dysentery and very severe febrile disease.

We used a low-cost, non-invasive technique (Liu *et al*. 1999; Khennavong *et al*. 2011) to determine how many children attending an outpatient department in Cambodia had evidence of antibacterial activity in their urine, suggesting recent antibiotic use. Our study additionally and uniquely, examined the presence of antibacterial activity against locally important resistant bacteria previously isolated from blood cultures in our hospital.

## Methods

### Location and participants

Angkor Hospital for Children (AHC) is a charitably funded 50-bed facility that serves as a referral paediatric hospital in Siem Reap Province, Cambodia. It is one of two referral hospitals serving the paediatric population of Siem Reap city and province. All paediatric patients younger than 16 years attending the outpatient department between 27 June 2011 and 5 July 2011, were eligible for inclusion (excluding 3rd July as there is no outpatient service on Sundays). Provision of a urine sample and written informed caregiver consent was necessary for participation in the study. Samples were stored at −4 °C for <8 h and divided into aliquots stored at −80 °C while awaiting batch testing within 6 months. Caregivers were asked for demographic information and whether their child had symptoms of fever, cough or diarrhoea in the preceding 48-h period. They were also asked whether their child had received any medication within that 48-h period and if so which, and where it was acquired. Ethical approval for the study was granted by the AHC Institutional Review Board.

### Laboratory procedures

The bacteria used in the initial screening assay were *Escherichia coli* (ATCC 25922), *Bacillus stearothermophilus* (ATCC 7953) and *Streptococcus pyogenes* (ATCC 19615). Each organism was prepared to a 0.5 Macfarland concentration and spread onto the agar in Petri dishes and tested as previously described (Khennavong *et al*. 2011). Mueller-Hinton agar was used as substrate for the *E. coli* and *B. stearothermophilus* isolates, and Mueller-Hinton agar with 5% sheep blood for the *S. pyogenes* isolate. Six 6-mm diameter discs of filter paper were placed onto three Petri dishes each containing one of the three screening assay isolates. Five microlitres of thawed urine was placed onto one disc per assay plate. This was performed in duplicate for each sample. The plates were incubated for 24 h at 35 °C for *E. coli* and *S. pyogenes* and at 56 °C for *B. stearothermophilus*. The zone diameter of inhibited growth around each disc was determined using a ruler. Identical results between the duplicates were recorded, but divergent results were repeated. The presence of any zone of inhibited growth defined the presence of urinary antibiotic activity. Quality control of the organisms and media was performed for each batch of samples as described previously (Khennavong *et al*. 2011).

Urine samples displaying antibiotic activity were further tested using the same methods. These samples alone underwent a second freeze-thaw cycle. They were tested for antibiotic activity against three resistant bacteria isolated from blood cultures from Cambodian children at AHC over the preceding 3 years: a methicillin-resistant *S. aureus* (MRSA) resistant to penicillin, erythromycin, gentamicin and ciprofloxacin and susceptible to trimethoprim–sulfamethoxazole and rifampicin using the CLSI disc diffusion standards (CLSI 2011); a *Salmonella enterica* serovar Typhi resistant to ampicillin, trimethoprim–sulfamethoxazole, chloramphenicol, nalidixic acid and ciprofloxacin (minimum inhibitory concentration = 0.25 mg/l) and susceptible to ceftriaxone; and an extended-spectrum *β*-lactamase-producing (ESBL-producing) *E. coli*, which was resistant to ampicillin, co-amoxiclav, chloramphenicol, trimethoprim–sulfamethoxazole, ciprofloxacin, gentamicin, ceftriaxone, ceftazidime and cefpodoxime and susceptible to imipenem and colistin.

## Results

During the study period, 3529 children attended the outpatients department and 775 (22%) children were included in the study. Their median (interquartile range) age was 4.6 years (2.5–8). 433 (56.7%) enrolled children were male (data missing for 11 children), and 581 (75.3%) children came from Siem Reap Province (data missing for 14 children). During the 2 days prior to attending outpatient department caregivers reported, 75.1% of children had a fever (582/775), 65.0% had a cough (504/775), and 24.3% had diarrhoea (188/775).

69% (535/775) of caregivers had given their child at least one medication in the 48 h preceding presentation. Of these, 392 gave a single preparation, 123 gave two, 18 gave three, and two gave four (Table1). 7.3% (57/775) of caregivers gave a known and named antibiotic; 37.8% (293/775) children were given one or more unknown medications. At least seven children were taking more than one paracetamol-containing preparation.

**Table 1:** Medications given in preceding 48 h (caregiver reported)

Antibiotics	
Amoxicillin	40
Ampicillin	5
Augmentin	4
Cephalexin	2
Cloxacillin	1
Co-trimoxazole	1
Erythromycin	1
Tuberculosis treatment	2
Tetracycline	2
Medication for chronic disease	13
Compound medications (containing paracetamol)*	9
Paracetamol	250
Miscellaneous†	42
Traditional	2
Unknown	326

* Compound preparations including an antihistamine, paracetamol and a decongestant.

† Anti-helminth (1), antihistamine (3), eye drops (1), hyoscine (1), loperamide (1), nose drops (3), IV fluids (4), oral rehydration salts (5), vitamins, folic acid or zinc (23).

The most common source for obtaining medications was a private pharmacy (251/558; 45%), followed by AHC (117, 21.0%), other health centres (100, 17.9%) and sources such as relatives, mobile ‘nurses’ or charities (28, 5.0%).

As the urinary assays (Table2) revealed, 31.7% (246/775) of urine samples showed antibiotic activity. Children with urinary antibiotic activity were more likely to have had a history of fever [odds ratio (OR) 3.4, 95% confidence intervals (CI) 2.2–5.2] or cough (OR 1.8, 95% CI 1.3–2.6) in the preceding 48 h, but not diarrhoea (OR 1.04, 95% CI 0.7–1.5). These children were also more likely to have been given a known antibiotic (OR 4.9, 95% CI 2.8–8.7) or any medicine (OR 9.2, 95% CI 5.5–15.3). Years of age were inversely related to urinary antibiotic activity (*P* = 0.004). When adjusted for history of any medicine intake, the relationships between cough, fever and age were weakened but remained statistically significant (*P* < 0.05 for all).

**Table 2:** Antibiotic activity in urine against screening assay bacterial isolates and resistant bacterial isolates

	Screening assay isolates*			
	*Escherichia coli*	*Bacillus* spp.	*Streptococcus pyogenes*	Any screening isolate
Number of children with urinary antibiotic activity to organism	69/775 (8.9%)	239/775 (30.8%)	182/775 (23.5%)	246/775 (31.7%)

	Resistant bacterial isolates†			
	*S. *Typhi	Methicillin-resistant *Staphylococcus aureus*	Extended-spectrum *β*-lactamase-producing *E. coli*	Any resistant isolate
Number of children with urinary antibiotic activity to organism	26/246 (10.6%)	15/246 (6.1%)	0/246	40/246 (16.3%)

* Sensitive strains used are as previously described: *E. coli* ATCC 25922, *Bacillus stearothermophilus* ATCC 7953 and *S. pyogenes* ATCC 19615.

† Resistant strains were isolated from bloodstream infections from paediatric patients admitted to the Angkor Hospital for Children.

16.3% (40/246) of urine samples that showed antibiotic activity did so against the MRSA or *S. typhi* isolates. No sample produced a zone of inhibition to the ESBL-producing isolate.

## Discussion

We showed that antibiotic use is common amongst paediatric outpatients in Cambodia, as it is in Laos (Khennavong *et al*. 2011), and other developing countries (Kotwani & Holloway 2011; Morgan *et al*. 2011). We may have underestimated usage as this method depends on active metabolites in the urine, which are dependent on pharmacokinetic factors (Driscoll *et al*. 2012) that were beyond the scope of this study. Other potential limitations are loss of activity during freezing and thawing of the samples and the possibility that other agents present in the urine may exert antibacterial activity (Khennavong *et al*. 2011).

A further limitation is that only 22% of children who attended as outpatients were included in the study and we cannot be sure that they were a truly random representative sample of paediatric outpatients in the hospital or of the healthcare-seeking child in the general population (particularly with another paediatric hospital in the region). Children in the study were not selected by symptoms or presenting complaint, and inclusion was solely based on whether the child was able to provide a urine sample and consent to participate being given. Caregiver reports of infective symptoms were common, suggesting the included children were more likely to have had an infection. We may therefore have overestimated antibiotic usage in the general paediatric outpatient population as children with suspected infection are most likely to have received an antibiotic. Against this, by including all comers, regardless of presenting complaint, we underestimate rates of antibiotic use in children with infective symptoms. As these children were anonymised, we do not know whether any were subsequently diagnosed with bacterial infections (in which case the use of antibiotics may have been appropriate) or whether a resistant organism was cultured. This would be an interesting focus for future work.

The demonstration of zones of inhibition to local MRSA and *S. typhi* isolates suggests that broad-spectrum ‘second-line’ antibiotics are being used in the community. However, the absence of urinary activity against an ESBL-producing *E. coli* suggests that carbapenems are not yet in widespread community use.

We are concerned that caregivers were administering unknown (often multiple) medications to their children, risking inadvertent toxicity from excessive dosing and drug interactions. This highlights the need for better education and regulation of pharmacists, better packaging in the local language and the importance of literacy and empowerment of caregivers. Our study demonstrates the need for community-based surveillance of both antibiotic use and resistance in Cambodia (WHO 2009), an essential component of the antibiotic stewardship interventions needed to mitigate the threat of antibiotic resistance.
